# Complete Genome Sequence of *Aerococcus urinaeequi* Strain AV208

**DOI:** 10.1128/genomeA.01218-16

**Published:** 2016-10-27

**Authors:** Wanqing Zhou, Dongmei Niu, Zhifeng Zhang, Yuan Liu, Mingzhe Ning, Xiaoli Cao, Chunni Zhang, Han Shen

**Affiliations:** a Department of Laboratory Medicine, Nanjing Drum Tower Hospital, the Affiliated Hospital of Nanjing University Medical School, Nanjing, People’s Republic of China; b Department of Clinical Laboratory, Jinling Hospital, Nanjing University School of Medicine, Nanjing University, Nanjing, People’s Republic of China

## Abstract

*Aerococcus urinaeequi* strain AV208 was isolated from an ascites sample from a patient with chronic kidney disease. The assembled genome contained 2,227,638 bp with a 39.1% G+C content. The genome harbors a *Tn1546* transposon-like structure with a *vanA* gene causing vancomycin resistance phenotypes of strain AV208.

## GENOME ANNOUNCEMENT

The genus *Aerococcus* was created by Williams et al. (1) to accommodate some Gram-positive, microaerophilic, catalase-negative organisms that were clearly distinguishable from streptococci and widely distributed in air, soil, milk, etc. (1). To date, eight species are included in the genus (2). They are described as probable opportunistic pathogens and can be isolated from a variety of clinical specimens including blood cultures of patients with subbacterial endocarditis, urine cultures of patients with urinary tract infections, synovial fluid cultures of septic arthritis, blood cultures of patients with bacteremia, and cerebrospinal fluid (CSF) cultures of patients with meningitis (3). To date, six *Aerococcus*-type strains have been whole-genome sequenced and published in NCBI (National Center for Biotechnology Information) (4). Here, we report the complete genome sequence of a vancomycin resistant *Aerococcus urinaeequi* strain which was incorrectly identified as *Aerococcus viridans* through phenotypic methods, isolated from peritoneal-related ascites from a 37-year-old woman with chronic kidney disease (5).

Genomic DNA was prepared using the QIAamp DNA minikit (Qiagen, Hilden, Germany) and was subjected to whole-genome sequencing using the Ion Torrent personal genome machine (Life Technologies, USA). Library construction and sequencing reactions were performed according to the manufacturer’s instructions and a 300 pair-end library was generated. The resulting sequences were *de novo* assembled using SOAPdenovo v2.01 (http://soap.genomics.org.cn/) (6). For the prokaryotic organism, we used an *ab initio* prediction method to get gene models for strain AV208. Gene models were identified using Glimmer 3 (7). Then, all gene models were blasted against the nonredundant (nr), SwissProt (http://uniprot.org), KEGG (http://www.genome.jp/kegg/) (8), and COG (http://www.ncbi.nlm.nih.gov/COG) (9) databases to do functional annotation. In addition, tRNA was identified using tRNAscan-SE, v1.23 (http://lowelab.ucsc.edu/tRNAscan-SE) (10) and rRNA (11) was determined using RNAmmer, v1.2 (http://www.cbs.dtu.dk/services/RNAmmer/).

The draft whole-genome sequence is composed of 2,227,638 bp with a 39.1% G+C content. The sequence resulted in 94 scaffolds with the largest scaffold of 107,388 bp. The *N*_50_ scaffolding size was 52,815. The assembled sequence reveals 1,990 protein coding genes, along with 28 tRNAs and eight rRNAs. These were single-copy genes predicted for 5S rRNA and 23S rRNA, and six duplicated genes predicted for 16S rRNA. We also identified putative antimicrobial resistance genes by comparing with the ARDB database. *ermt*, *aac6*, and *vanA* that show resistance to erythromycin, aminoglycoside antibiotics, and glycopeptide were found in the AV208 strain. Additionally, a *Tn1546* transposon-like structure that contains *transposase*, *vanR*, *vanS*, *vanH*, *vanA*, *vanX*, and *vanY* was located in scaffold 38 in the genome of strain AV208. This was consistent with the susceptibility of vancomycin and teicoplanin, which showed an *E* test result of >256 µg/mL. Comparisons with *A. urinaeequi* CCUG 28094^T^ and *A. urinaeequi* USDA-ARS-USMARC-56713 genomes revealed that the AV208 strain shares 1,489 and 1,483 orthologous coding sequences with *A. urinaeequi* CCUG 28094^T^ and *A. urinaeequi* USDA-ARS-USMARC-56713, respectively.

In conclusion, we presented the complete genome sequence of *A. urinaeequi* strain AV208. Further genome analysis of *A. urinaeequi* strain AV208 that shows vancomycin resistance will allow a better understanding of the resistance mechanisms.

### Accession number(s).

This whole-genome shotgun project has been deposited at DDBJ/ENA/GenBank under the accession no. MDRY00000000. The version described in this paper is MDRY01000000.
